# Effects of robot assisted gait training in progressive supranuclear palsy (PSP): a preliminary report

**DOI:** 10.3389/fnhum.2014.00207

**Published:** 2014-04-17

**Authors:** Patrizio Sale, Fabrizio Stocchi, Daniele Galafate, Maria Francesca De Pandis, Domenica Le Pera, Ivan Sova, Manuela Galli, Calogero Foti, Marco Franceschini

**Affiliations:** ^1^Department of Neurorehabilitation, IRCCS San Raffaele PisanaRome, Italy; ^2^San Raffaele CassinoCassino, Italy; ^3^Dipartimento di Elettronica, Informazione e Bioingegneria-Politecnico di MilanoMilano, Italy; ^4^Physical Rehabilitation Medicine Chair, Clinical Sciences and Translational Medicine DPT, Tor Vergata UniversityRoma, Italy

**Keywords:** progressive supranuclear palsy, PSP, gait analysis, lower limb, robot

## Abstract

**Background and Purpose:** Progressive supranuclear palsy (PSP) is a rare neurodegenerative disease clinically characterized by prominent axial extrapyramidal motor symptoms with frequent falls. Over the last years the introduction of robotic technologies to recover lower limb function has been greatly employed in the rehabilitative practice. This observational trial is aimed at investigating the changes in the main spatiotemporal following end-effector robot training in people with PSP.

**Method:** Pilot observational trial.

**Participants:** Five cognitively intact participants with PSP and gait disorders.

**Interventions:** Patients were submitted to a rehabilitative program of robot-assisted walking sessions for 45 min, 5 times a week for 4 weeks.

**Main outcome measures:** The spatiotemporal parameters at the beginning (T0) and at the end of treatment (T1) were recorded by a gait analysis laboratory.

**Results:** Robot training was feasible, acceptable and safe and all participants completed the prescribed training sessions. All patients showed an improvement in the gait spatiotemporal index (Mean velocity, Cadence, Step length, and Step width) (T0 vs. T1).

**Conclusions:** Robot training is a feasible and safe form of rehabilitation for cognitively intact people with PSP. The lack of side effects and the positive results in the gait parameter index in all patients support the recommendation to extend the trials of this treatment. Further investigation regarding the effectiveness of robot training in time is necessary.

**Trial registration:** ClinicalTrials.gov NCT01668407.

## Introduction

Progressive supranuclear palsy (PSP) is a rare neurodegenerative disease that causes the gradual deterioration and death of specific volumes of the brain (Richardson et al., 1963; Steele et al., 1964). Ludolph and colleagues showed that the neuropathologic features of PSP include marked midbrain atrophy and atrophy of the pallidum, thalamus, subthalamic nucleus, and frontal lobes (Ludolph et al., 2009). Clinical criteria for the diagnosis of PSP are: a gradually progressive disorder, an onset at the age of 40 years or later, a presence of vertical supranuclear palsy, a slowing of vertical saccades and a postural instability with falls in the first year of disease onset. In particular, the first symptoms in two-thirds of the cases are: loss of balance, lunging forward when mobilizing, fast walking, bumping into objects or people, and falls (Lubarsky and Juncos, 2008). Other common early symptoms are changes in personality, general slowing of movement and visual symptoms. Postural instability and gait impairment are the most important disorders in the early phases of the disease. The PSP subject shows a short, shuffling stepped gait, gait freezing, lurching, unsteady gait or spontaneous falls (Lubarsky and Juncos, 2008). In particular, subjects with PSP have decreased step length, step velocity, and a significantly slower ability to break a fall from the center of gravity than controls (Welter et al., 2007). Although some disorders are similar to those found in Parkinsonism (rigidity, bradykinesia, postural instability), PSP differs in several ways. The PSP subject does not have a forward flexed posture like the Parkinson Disease (PD) subject and falls in PSP tend to be backwards (Boeve, 2007). Pharmacotherapy with carbidopa/levodopa and with dopamine agonists typically is ineffective in managing the disorders (Rampello et al., 2005). Non-pharmacologic therapies such as physical therapy and occupational therapy are potentially useful, with the main goals being the maintenance of functional ambulation and the reduction of falls and associated injuries (Steffen et al., 2007). Gait recovery in all patients with impairments of the central nervous system (CNS) is an integral part of rehabilitation and often influences the possibility of a patient returning home or to work (Schmidt et al., 2007). Neurologic motor rehabilitation is directed toward the re-learning of motor skills. In particular, the recovery of walking is a crucial aspect of rehabilitation, improving the quality of life and patient's independence. For non-ambulatory neurological patients, mechanically assisted walking with body weight support (BWS) has been suggested as a strategy to facilitate walking (Richards et al., 1993; Hesse et al., 2001) because it provides the opportunity to complete more practice of the whole task than would be possible by assisting overground walking. In the last years, robot-aided walking is considered a promising tool for gait rehabilitation (Colombo et al., 2000) in various neurological disease (Beer et al., 2008; Lo and Triche, 2008; Hesse et al., 2010). The robotic devices have been developed to relieve physical therapists from the strenuous and not ergonomic burden of manual BWS (Volpe et al., 2001; Winchester and Querry, 2006; Steffen et al., 2007). Furthermore, the use of robotic devices is currently advised to prevent the risk of falls and to improve gait velocity with the total safety of the patient (Morone et al., 2014). Moreover, robot devices can be used to give inpatients an intensive program (in terms of many repetitions) of complex gait cycles. The robotic machines, offering practice up to 1000 steps per session, used either an exoskeleton or an end-effector approach. Currently, a robotic task-specific repetitive approach, i.e., numerous practices of complex gait cycles, is regarded as the most promising to restore motor function after neurological disease (Dobkin, 2004; Dobkin and Duncan, 2012). Evidence-based approaches of rehabilitation in PSP are lacking and currently the majority of research on these subjects consists of case reports involving only a small number of patients. Until now, only few studies have been carried out on physiotherapy intervention in the PSP gait and balance disorder (Izzo et al., 1986; Sosner et al., 1993; Suteerawattananon et al., 2002; Steffen et al., 2014). Usually the subject with PSP needs rehabilitative training to improve balance and gait problems and to prevent the frequent falls. The rehabilitation programs for patients with PSP generally include limb-coordination activities, tilt-board balancing, gait training, strength training with progressive resistive exercises and isokinetic exercises and stretching of the neck muscles (Miyai et al., 2002). Even if several studies have demonstrated the efficacy of workout, including treadmill training, in subjects with PD (Zampieri and Di Fabio, 2006; Semprini et al., 2009; Mehrholz et al., 2010), so far little evidence has been found on the effects of the treadmill training therapy on tauopathies or other parkinsonian disorders (Suteerawattananon et al., 2002; Steffen et al., 2007) and no study has been conducted on the employment of robot assisted treatment in PSP subjects. This study was aimed at investigating the effects on improvement of the walking function by a change in spatiotemporal parameters using end-effector robotic rehabilitation locomotion training in patients with PSP.

## Materials and methods

This study was a pilot observational trial. The subject had been on stable doses of medication for at least 4 weeks prior to study onset and showed an endurance sufficient to keep an upright position, assisted or unassisted, for at least 15 min. A preliminary medical examination included a physical and neurological test besides a gait analysis. The inclusion criteria for all groups were: (a) diagnosis of idiopathic PSP according to the UK Brain Bank criteria, without any other significant neurological or orthopaedic disorder; (b) age between 18 and 90 years old; (c) ability to walk, unassisted or with little assistance, for at least 25 feet. The following exclusion criteria were identified: (d) inability to understand instructions required by the study (Informed Consent Test of Comprehension); (e) primarily wheelchair bound; (f) chronic and ongoing alcohol or drug abuse, active depression, anxiety or psychosis that might interfere with the use of the equipment or testing.

## Instrumental assessments

Trained professionals, who were not involved in the research treatment and blind to patients' treatment, performed all instrumental and clinical assessments. All outcome assessments were collected in the ON phase 1 h and half after oral assumption of the usual dose of medication. The 3D-Gait analysis (3D-GA) was conducted using the following equipment: a 12-camera optoelectronic system with passive markers (ELITE2002, BTS, Italy) to measure the kinematics of the movement; 2 force platforms (Kistler, CH) to obtain the kinetic data of the movement (i.e., ground reaction forces); 2 TV camera Video systems (BTS, Italy) synchronized with the optoelectronic and force platform systems for video recording. To evaluate the kinematics of each body segment, markers were positioned as described by Davis and colleagues (Sale et al., 2012, 2013a). Subjects were asked to walk barefoot, at their own natural pace (self-selected and comfortable speed), along a 10-meter walkway where the two force platforms were placed. At least seven trials were collected for each subject in order to ensure data consistency. All graphs obtained from GA were normalized as a % of the gait cycle and kinetic data were normalized for individual body weights. In order to quantify the gait pattern of participants involved in this study, a specific software (Smartanalyser, BTS, Italy) enabled the calculation of some indices (time/distance parameters, joint angles values in specific gait cycle instant, peak values in ankle power graph) starting from those data.

## Primary outcomes

A primary outcome was the change in spatiotemporal parameter. In particular we recorded: the gait velocity assessed by mean velocity (m/s), which measured the rate of change of position, recorded in meters per second; the cadence (step/min) that measured the number of steps taken in a given period of time, which was then converted into the number of steps taken per minute; the step length (mm) that measured the average distance (mm) between two successive placements of the same foot; the stride length (mm) that measured the average distance (mm) between two successive placements of the same foot, the step width (mm) that measured the medio-lateral distance between the two feet during double support; the stance time (stride %) that measured the duration of the stance phase; the swing time (stride %) that measured the duration of the swing phase and the double support (stride %) that measured the duration of double support.

## Robot therapy

Each subject was asked to perform 20 sessions (5 days a week for 4 weeks) of robot assisted gait training, using the commercially available end effector system machines G-EO system device (Reha Technology AG; Olten, Switzerland). The trajectories of the footplates and the vertical and horizontal movements of the center of mass were fully programmable, enabling wheelchair-bound subjects not only the repetitive practice of simulated floor walking but also up and down stair climbing. One therapist, who has experience in machine-supported gait rehabilitation, assisted the patients with putting on the harness while sitting in their wheelchair, getting onto the G-EO System in the wheelchair using a ramp from the rear, fixing the feet on the plates, hoisting the patient, attaching the lateral ropes, and setting the therapy parameters memorized by the G-EO System computer. The footplates had 3 DoF each, allowing to control the length and the height of the steps and the foot plate angles. The maximum step length corresponded to 550 mm, the maximum achievable height of the steps was 400 mm and the maximum angles were ±90°. The maximum speed of the foot plates was 2.3 km/h. The exercise included a robot-assisted walking therapy, at variable speeds, for a maximum of 45 min, with a partial BWS. All participants started with 30–40% of BWS and an initial speed of 1.5 km/h; speed was then increased to a range of 2.2–2.5 km/h maximum and initial BWS was decreased. Two further DoF controlled the BWS system and the lateral displacement of the hip. The graphic user interface (GUI) showed during the training the actual trajectory, so that the therapists were able to control and to correct it. Changes during the training were made for step length, step height, the terminal stance and the initial contact inclination angles of the feet, the vertical and the lateral excursions of the CoM, and for the relative position of the suspension point with respect to the footplates. The computer saved the trajectory settings. The adaptive control was applied only to both of the 3 DoF intended to control the legs. The remaining 2 DoF for the control of the center of mass were excluded from being master in the adaptive mode. In particular, the patients must apply the necessary force for moving the footplates along the selected trajectory. The necessary force for moving the footplates along the selected trajectory settings was set by a force level slider in the software of the robot. The computer put the footplates and the mechanics of the G-EO Systems to virtual zero friction and once the intended force level on the lower limb was reached, there could be amplification to the movement, according to the value of the amplification level selected in the robot by therapist. The amplification level provided for additional acceleration while executing the movement of the footplates along the selected trajectory. By adding acceleration to the necessary force for moving, the footplates lowered. This provided a smooth and continuous movement of the footplates. After 45 min the session was stopped. During each session, the patients practiced 5–30 min of simulated floor walking followed by 5 to 10 min of repetitive simulated stair climbing up and down. The patient practiced a minimum of 300 steps on the simulated floor and climbed a minimum of 50 steps on the simulated stair during each session. Breaks were optional, but uninterrupted training intervals of at least 5 min for simulated floor walking and 3 min of simulated stair climbing were required. Heart rate and blood pressure were monitored at the beginning and at the end of each session. Subjects who did not retrieve sessions and interrupted the treatment for more than 3 consecutive days were excluded from the study.

## Statistical analysis

All the previously defined parameters were computed for each participant. Mean values and standard deviations of all indexes were calculated for each group. The Kolomogorov–Smirnov tests were used to verify if the parameters were normally distributed. As this was not the case, we used Wilcoxon's tests in order to detect significant changes between data at baseline (T0) and endpoint (T1). The T0 and T1 data of all patients and CG were compared with Mann-Whitney *U* tests. Statistical significance was set at *p* < 0.05. The Mann-Whitney test was used to compare median scores between groups.

## Ethical aspects

This study was performed in accordance with the Declaration of Helsinki and was approved by the ethics committees of IRCCS San Raffaele Pisana. Informed consent was obtained from all subjects enrolled in this study.

## Results

We screened 15 patients, 5 of whom satisfied the inclusion criteria. No dropouts were recorded during the treatment and all subjects fulfilled the protocol (compliant subjects: *N* = 5). The distribution of the study subjects (*N* = 5) by age, gender, and main clinical and demographical characteristics are shown in Table 1. The median age was 67.80 ± 11.71 years. Participant demographics are presented in Table 1. Table 2 summarizes the observed mean ± SD for all tests (T0 vs. T1), as measured on the compliant subjects at T0 (*N* = 5), T1 (*N* = 5) (Table 2). Gait velocity (T0 0.54 ± 0.173 m/s and T1 0.69 ± 0.150 m/s) and cadence (T0 83.00 ± 9.618 and T1 93.60 ± 15.437) improved respectively by 15 and 23.8%. Participants also demonstrated an improvement of 11% in step length left (T0 421.00 ± 98.831 mm and T1 466.40 ± 105.749 mm) and of 35% in step length right (T0 363.20 ± 94.767 mm and T1 429.80 ± 67.570 mm) and a decrease of 9% of Step width (T0 166.60 ± 24.460 mm and T1 153.60 ± 43.678 mm) (Table 2). Due to a small analyzed sample size no statistical significance was found in all the analyzed parameters. No significant changes were found in SpO2 (T0 97.5 ± 1.6 T1 97 ± 4.1, and in the HR (T0 82.3 ± 13.1 T1 86.9 ± 16) after the trainings in all patients. The patients workload and statisfaction assessed by a Visual Analogical Scale showed a tendency toward positive feelings regarding the training process. Moreover regarding the fatigue the patients reported that during and at the end of the training training there was no excessive fatigue. The patients did clearly feel safe and comfortable with the robot at the end of the training.

**Table 1:** **Patients' clinic and demographic data at baseline**.

**Experimental group (*n* = 5)**		
	***n***	***Mean ± SD***
Dropout	0	
Compliants	5	
Male	3	
Female	2	
Age		67.8 ± 11.7
Height		161.2 ± 3.56
Weight		72.00 ± 10.58
Years of disease		3.6 ± 1.85
Psp rating scale		32.00 ± 9.24
PSP staging scale		2.4 ± 0.5
Walking handicap scale		3.2 ± 1.48

Legend n, number; SD, standard deviation

**Table 2:** **Results of gait analysis spatiotemporal parameters of all single patients and mean at T0 and T1**.

		**Sex**	**Età**	**Altezza**	**Peso**	**T0 Mean velocity (m/s)**	**T1 Mean velocity (m/s)**	**T0 Cadence (step/min)**	**T1 Cadence (step/min)**	**T0 Step length (mm) DX**	**T1 Step length (mm) DX**	**T0 Step length (mm) SX**	**T1 Step length (mm) SX**	**T0 Step width (mm)**	**T1 Step width (mm)**
	P1	Female	52	158	70	0.53	0.68	91	113	349	384	353	344	173	165
	P2	Female	68	165	68	0.49	0.62	70	72	328	471	492	562	156	143
	P3	Male	78	158	70	0.43	0.57	77	86	349	410	336	400	205	220
	P4	Male	80	160	62	0.42	0.63	84	97	268	359	365	436	159	101
	P5	Female	61	165	90	0.84	0.95	93	100	522	525	559	590	140	139
Mean			67.80	161.20	72.00	0.54	0.69	83.00	93.60	363.20	429.80	421.00	466.40	166.60	153.60
*SD*			11.71	3.56	10.58	0.173	0.150	9.618	15.437	94.767	67.570	98.831	105.749	24.460	43.678
		**Sex**				**T0 Stance time (% stride) DX**	**T1 Stance time (% stride) DX**	**T0 Stance time (% stride) SX**	**T1 Stance time (% stride) SX**	**T0 Double supp. (% stride) DX**	**T1 Double supp. (% stride) DX**	**TO Double supp. (% stride) SX**	**T1 Double supp. (% stride) SX**		
	P1	Female				62%	63%	62%	63%	14%	14%	12%	11%		
	P2	Female				60%	63%	71%	62%	19%	17%	11%	8%		
	P3	Male				62%	65%	58%	61%	14%	15%	10%	9%		
	P4	Male				64%	60%	68%	57%	15%	8%	14%	8%		
	P5	Female				60%	59%	59%	57%	13%	11%	6%	6%		
Mean						62%	62%	64%	60%	15%	13%	11%	8%		
*SD*						0.017	0.024	0.057	0.028	0.023	0.035	0.030	0.018		

## Discussion

Many authors have shown the efficacy of robot-assisted gait training on improving the walking function in several neurological diagnoses (Wirz et al., 2005; Morone et al., 2012; Schwartz et al., 2012; Sale et al., 2013a) but the process aimed at restoring this function in patients with a neurological pathology is challenged by the complexity and variability of these disorders (Sale et al., 2012). As far as we know, this is the first study that examines the effects of end effector robot-assisted training in individuals with PSP. This study shows that twenty 45-min sessions of robot-assisted training is a treatment that could stimulate and enhance the beneficial effects of motor training on gait recovery in patients with PSP. This trial protocol is easy, reproducible and safe and it allows the training of PSP subjects with moderate to severe lower limb impairment. As demonstrated, in gait training to walk repetitively in a natural manner similar to the over-ground gait and with the correct proprioceptive and exteroceptive feedback is of the most critical importance (Sale et al., 2012). In particular our adaptive training robotic protocol where the patient interacts with the robot can overcome all the limitations about the repetitively (and repeatability) of the movement with respect to the human-human interaction. Until now, several studies have demonstrated that robotic gait training can slow the clinical progression of gait disease in PD patients, but so far, no other evidence has been found in the PSP population. In particular, robot-assisted training may improve postural instability in patients with PD (Picelli et al., 2012a) or may develop aspects of walking ability (Picelli et al., 2012b). In our recent paper we demonstrated that the use of the end-effector lower limb robotic device in PD patients increases a short period lower limb motor recovery in idiopathic PD patients, improving above all the gait velocity, the step length and the stride length (Sale et al., 2013b). To date, there are not many studies on the gait rehabilitation in patients with PSP. Recently Steffen and colleagues published an interesting case report on the use of the treadmill in gait recovery in one patient with PSP, with a follow-up of 10 years. In particular, the author showed how a patient with PSP participated consistently in a regular group exercise program for 10 years and how he reduced fall frequency, maintained balance and endurance and retained community ambulation using a walker (Steffen et al., 2014). Falls and the associated trauma are one of the main causes of morbidity and mortality in these disorders and the lower limb training could effectively prevent it; falls are also the leading cause of unintentional injury and hospitalization in people aged 65 years and older (Dellinger and Stevens, 2006; Bridenbaugh and Kressig, 2011). Different methods of gait rehabilitation have been used so far in neurological lower limb physiotherapy to prevent the falls, such as manually assisted over-ground training and manually assisted treadmill training or robotic training with or without the BWS (Sale et al., 2012). The advantage of these electromechanical devices, compared with treadmill training with partial BWS in PD and Stroke patients, may be the reduced effort required by therapists, as they no longer need to set the paretic limbs or assist trunk movements (Hesse et al., 2003). However, the effort in the development of robotic gait training is not limited to the improvement of working conditions of physiotherapists, but rather in providing the patient with an engaging, challenging and effective rehabilitation tool. Our choice to use the robotic device in PSP gait recovery has many bases. Our training shows in all patients an improvement in each spatio-temporal parameter, in particular cadence, step length, stride length, velocity, and reduction of step width. These are the parameters mostly connected to the risk of falls. In particular, several studies have identified changes in certain spatial and temporal gait parameters as independent predictors of the fall risk. Tailored and colleagues showed in a sample of older people that there were significant main effects of gait condition and of faller status for mean value measures (velocity, stride length, double support time, and stride width) and for variability measures (swing time variability and stride length variability); the examination of individual gait parameters indicated that the multiple fallers walked more slowly, had a shorter stride length, spent more time in double support, had a wider support width and showed more variability in stride length and swing time (Taylor et al., 2013). Maki observed in his study that increased stride-to-stride variability in stride length, stride speed and double support time as well as increased stride width were predictive of falls in the ensuing 6 months for residents of a senior living facility (Maki, 1997). Our data, compared with the results of Tailored and Maki, suggest that the robot therapy, with the intention to recover the leg movements, could increase and help these patients to prevent falls and other trauma. Several conditions should however be considered when interpreting these data. Most importantly, these are simply case descriptions. Changes in these 5 individuals could have been due to a variety of factors. Possibly the improvements could represent progresses in test performance. Or perhaps the observed changes could be due to usual day-to-day, but it should be noted that the trends in the repeated measures were fairly consistent and showed improvement in all patients. A long period follow-up is required to confirm our hypothesis. Furthermore, it is unknown to what extent these findings will generalize in other individuals in the early and middle stages of PSP. These 5 individuals were in the early to middle stages of PSP and highly motivated, which may have contributed to their reported adherence, even after completing the supervised part of the program. Our experience and various examined articles showed that robot-assisted gait therapy provides versatile control approaches as a framework in the design of optimal rehabilitation interventions and experimental motor control studies, but the high cost of robot devices raises the question of efficiency in comparison with other training strategies.

## Conclusion

The focus on gait recovery represents one of the most innovative features of this study and makes this research useful in clinical practice. Spatial-temporal gait analysis can detect discrete gait disorders, which are not perceptible to the naked eye, and several gait changes have been identified as fall predictors. Early detection allows early intervention. The positive results on improvement in spatiotemporal parameter of the PSP subject by the Robot Therapy, the lack of side effects strongly recommend extending the use of a Robot Therapy in the recovery of gait performance. This rehabilitation training could provide new opportunities in PSP rehabilitation thanks to a sensorimotor approach aimed at functional recovery.

## Author contributions

All authors contributed to the design of the study, to draft and review the manuscript. Patrizio Sale, Calogero Foti, Daniele Galafate, Domenica Le Pera contributed to data analysis. Patrizio Sale, Marco Franceschini, Maria Francesca De Pandis and Fabrizio Stocchi contributed in the supervision of the research study. All authors read and approved the final manuscript.

### Conflict of interest statement

The authors declare that the research was conducted in the absence of any commercial or financial relationships that could be construed as a potential conflict of interest.
